# Transcriptome Analysis Provides Insight into the Molecular Mechanisms Underlying *gametophyte factor 2*-Mediated Cross-Incompatibility in Maize

**DOI:** 10.3390/ijms19061757

**Published:** 2018-06-13

**Authors:** Man Wang, Zhibin Chen, Huairen Zhang, Huabang Chen, Xiquan Gao

**Affiliations:** 1 State Key Laboratory of Crop Genetics and Germplasm Enhancement, College of Agriculture, Nanjing Agricultural University, Nanjing 210095, China; 2014201002@njau.edu.cn; 2 Jiangsu Collaborative Innovation Center for Modern Crop Production, Nanjing Agricultural University, Nanjing 210095, China; 3 State Key Laboratory of Plant Cell and Chromosome Engineering, Institute of Genetics and Developmental Biology, Chinese Academy of Sciences, Beijing 100101, China; zbchen@genetics.ac.cn (Z.C.); hrzhang@genetics.ac.cn (H.Z.); 4 University of Chinese Academy of Sciences, Beijing 100049, China

**Keywords:** *Ga2*, unilateral cross-incompatibility, silk, pollen tube, transcriptome, *Zea mays* L.

## Abstract

In maize (*Zea mays* L.), unilateral cross-incompatibility (UCI) is controlled by *Gametophyte factors* (*Ga*), including *Ga1*, *Ga2*, and *Tcb1*; however, the molecular mechanisms underpinning this process remain unexplored. Here, we report the pollination phenotype of an inbred line, 511L, which carries a near-dominant *Ga2*-*S* allele. We performed a high-throughput RNA sequencing (RNA-Seq) analysis of the compatible and incompatible crosses between 511L and B73, to identify the transcriptomic differences associated with *Ga2*-mediated UCI. An in vivo kinetics analysis revealed that the growth of non-self pollen tubes was blocked at the early stages after pollination in 511L, maintaining the UCI barrier in *Ga2*. In total, 25,759 genes were expressed, of which, 2063 differentially expressed genes (DEGs) were induced by pollination (G_GG, G_GB, B_BB, B_BG). A gene ontology (GO) enrichment analysis revealed that these genes were specifically enriched in functions involved in cell wall strength and pectic product modification. Moreover, 1839, 4382, and 5041 genes were detected to differentially express under same pollination treatments, including B_G, BG_GG, and BB_GB, respectively. A total of 1467 DEGs were constitutively expressed between the two inbred lines following pollination treatments, which were enriched in metabolic processes, flavonoid biosynthesis, cysteine biosynthesis, and vacuole functions. Furthermore, we confirmed 14 DEGs related to cell wall modification and stress by qRT-PCR, which might be involved in *Ga2*-*S*-mediated UCI. Our results provide a comprehensive foundation for the molecular mechanisms involved in silks of UCI mediated by *Ga2*-*S*.

## 1. Introduction

Maize (*Zea mays* L.) is a typical cross-pollinating crop with distinct male and female flowers on the same plant. In general, both selfing and crossing strategies are readily successful in most maize lines; however, certain lines fail to set seeds when pollinated with non-self pollen, hybridizing in only one direction. The factors governing this unilateral cross-incompatibility (UCI) were initially designated *Gametophyte factors* (*Ga*) because of the involvement of the gametes in this selective fertilization [1,2,3,4,5]. Numerous *Ga* loci have been reported, among which *Ga1* [4], *Ga2* [6,7], and *Teosinte crossing barrier 1* (*Tcb1*) [8,9] are of particular interest, because they cause near-complete cross-incompatibility. There are three distinct *Ga* haplotypes: (1) *S*-haplotype (*Ga-S*), which exerts a female UCI function by producing a barrier to non-self pollen, while male functions are unaffected, and self-pollen can avoid the barrier; (2) *M*-haplotype (*Ga-M*), displaying only male UCI activities; and (3) *ga* (*ga* wild type), which lacks any UCI activity [10,11,12,13]. The majority of dent and flint maize varieties are *ga* types. During fertilization, *Ga-S* plants completely reject pollen from *ga* plants due to the female barrier, but accept pollen from *Ga-S* and *Ga-M* haplotypes, producing pollen compatible for selfing and reciprocal crosses; however, the *Ga1*, *Ga2*, and *Tcb1* alleles are cross-incompatible with each other.

*Ga1* and *Tcb1* were mapped to chromosome 4 [8,12,14,15,16], and *Ga2* is located on chromosome 5 [6,7,17]. Despite the extensive fine mapping efforts for male and female functions of *Ga1-S* locus, the regions identified for male and female functions have not yet been physically separated [12,15]. Using heteroallelic pollen, cross-incompatibility in *Ga1-S* was demonstrated to arise from pollen–pistil incongruity, rather than the active rejection of pollen by the pistil [18].

Any defect during pollen germination, entry of the pollen tube into the style, growth of the pollen tube in the transmitting tract, or double fertilization, could lead to cross-incompatibility [19,20]. Altered pollen tube growth rates and abnormal morphology were previously observed in incompatible silks [12,21,22]. The apical cell wall of the pollen tube displays dramatic growth features; pollen tubes grow quickly to deliver their sperm cells, requiring the constant delivery of new cell wall materials, and the maintenance of appropriate ion gradients at the pollen tube tip [23,24,25].

Many factors, including Ca^2+^, pH, pectin methylesterases (PMEs; EC3.1.1.11), and the PME inhibitor (PMEI), have been found to be involved in pollen tube growth processes. Numerous reports have shown that the Ca^2+^ gradients at the pollen tube growth tip are essential for polarized growth, cell wall formation, and pollen tube guidance [26,27,28,29,30]. Pectins are the major component of primary cell walls in plants, and are usually present in a highly methylesterified form. The growing apex of the pollen tube region is composed of single fibrillar pectin wall layer, whereas the bilateral cell wall consists of cellulose and callose [31]. PMEs are cell wall modification enzymes that catalyze the demethylesterification of the pectic polysaccharide homogalacturonan, releasing methanol and protons, and exposing carboxyl residues, which are crosslinked by Ca^2+^ to provide structural support to the cell wall [32]. As a result, the strength of the apical cell wall and the pH levels surrounding the growing pollen tube are dynamically regulated by the PMEs and other wall enzymes to retain proper internal turgor and promote rapid growth [33,34]. The catalytic activity of the PMEs can be regulated by the PMEIs, a class of proteinaceous inhibitor that forms a 1:1 non-covalent complex to inhibit PME activity [35,36]. Flower-specific expression of *PMEI* was found to impact anther development in *Brassica rapa*, leading to male sterility [37]. The overexpression of *ZmPMEI1* in female maize gametophytes destabilized the subapical cell wall of the pollen tubes and caused them to burst [38]. During pollen–pistil interactions, the pollen (or pollen tube) and pistil (or embryo sac) secrete cysteine-rich proteins and lipid transfer proteins to exert diverse functions [39,40], such as self-recognition in the self-incompatibility (SI) system via the male determinant S locus cysteine-rich protein (SCR) [41,42], or the guidance of female tissues to regulate pollen tube growth [43,44]. Intriguingly, other factors, such as rapid alkalinization factors (RALFs) 4 and 19, were reported to interact with the receptor-like kinase (RLK) *Catharanthus roseus* RLK1-like subfamily (CrRLK1L) to maintain pollen tube integrity [45,46]. Furthermore, the female-derived ligand RALF34 induced pollen tube burst and sperm release, indicating that this protein may function in relieving RALF4/19 suppression to facilitate double fertilization [47].

Plants control SI by diverse molecular mechanisms, such as the perception of peptide ligands by receptor kinases in the Brassicaceae [41,42], glycoprotein-mediated cell death in Papaveraceae [48,49], and RNase-mediated protein degradation systems in the Solanaceae, Rosaceae, and Plantaginaceae families [50,51]. Interestingly, similar to SI, UCI in Solanaceae is also mediated by a protein-degradation system regulated by pollen-expressed *Cullin1* and an F-box gene, *SLF-23* [52,53], suggesting that both SI and UCI likely deploy the same structures and products of the pistil and pollen to achieve fertilization, and conserved mechanisms mediated by certain genes may exist for both SI and UCI. However, the abovementioned mechanisms regulating SI/UCI are only found in dicotyledons, and it is unclear whether distinct mechanisms governing SI/UCI exist in monocot plants.

Although *ga1* was discovered in the early 1900s, no *Ga* has been successfully cloned. Moreover, the molecular and genetic mechanisms of *Ga*-mediated cross-incompatibility are largely unknown. In this study, we performed a transcriptomic analysis to investigate the global gene expression profiles in unpollinated and pollinated maize silks between compatible and incompatible crosses of the *Ga2-S* locus. We found that large numbers of differentially expressed genes (DEGs) exist between the cross-incompatibility inbred line 511L and the B73 cultivar, and that pollination induced the expression of genes involved in signal transduction, cell wall metabolism, and stress responses. Specifically, several candidate genes with functions in cell wall metabolism and receptor-like kinase recognition signaling were found to be associated with UCI in maize.

## 2. Results

### 2.1. Phenotypic and Genotypic Analysis of Ga2-S

**Figure 1:**
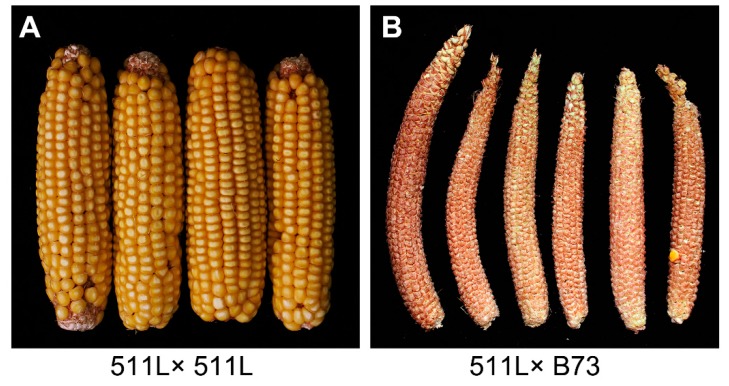
The phenotype resulting from the *gametophyte factor 2* pistil barrier in 511L. (**A**) Seed setting of selfed 511L; (**B**) Seed setting of 511L pollinated with B73 pollen (*n* = 30).

To investigate the role of *Ga2* in controlling cross-incompatibility, we obtained the 511L inbred line from the MaizeGDB stock center. To confirm its incompatibility, we initially crossed 511L as the female parent with ten Chinese inbred lines. None of the crosses set seeds, whereas selfing 511L resulted in seeds being set on the cob (Figure 1A). We then de-tasseled 30 511L plants, and pollinated them three times with B73 pollen. Of these crosses, 21 plants set no seeds, and the rest set 1–6 seeds per cob (Figure 1B), indicating that the cross-incompatibility of 511L is near-complete. We further examined the incompatibility of 511L (*Ga2-S*) by crossing it with SDGa25 (*Ga1-S*) [12,14]. Neither 511L × SDGa25 nor SDGa25 × 511L set seeds; thus, we confirmed that 511L contains an *S*-haplotype at the *Ga2* locus.

**Figure 2:**
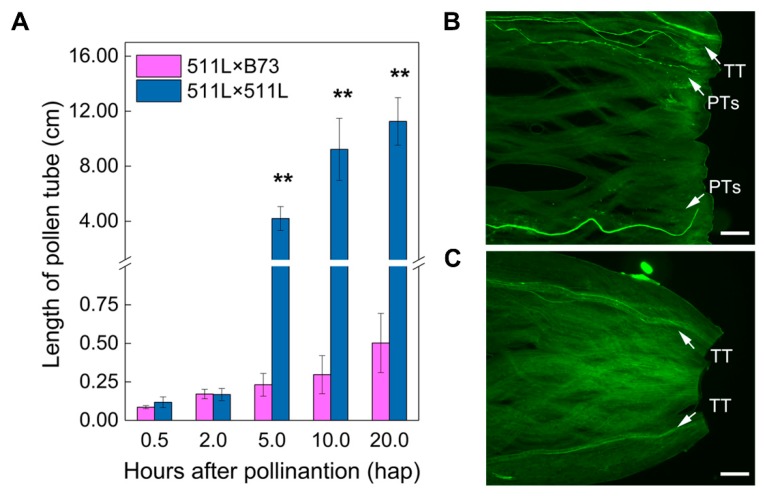
Kinetics and morphology of in vivo pollen tube growth in compatible (511L × 511L) and incompatible (511L × B73) cross combinations. (**A**) Error bars indicate the SD of the lengths of 30 pollen tubes. Asterisks indicate significant differences in comparison with the corresponding values in an incompatible cross (** *p* < 0.01, Student’s *t*-test); (**B**) 511L/511L 20 hap and (**C**) 511L/B73 20 hap. TT, transmitting tract; PTs, pollen tubes; Bar = 200 μm.

We next compared pollen germination and pollen tube growth between compatible (511L × 511L) and incompatible (511L × B73) crosses at different time points after pollination. The pollen germinated well in both crosses, and no significant differences in pollen tube length were observed between the compatible and incompatible crosses at 2 h after pollination (hap; Figure 2A). The pollen tubes in the compatible crosses grew rapidly (approximately 6 mm h^−1^) after 2 hap, with most pollen tubes reaching the silk base at 20 hap (Figure 2B). By contrast, the pollen in the incompatible cross grew at only 0.25 mm h^−1^, and growth had virtually stopped at 2 hap, and the pollen tubes rarely reached 0.5 cm in length (Figure 2A,C). The results suggested that cross-incompatibility between *Ga2-S* and *ga2* was caused by pollen tube growth arrest in the early stages following pollination.

### 2.2. Transcriptome Profiling of Silks from Compatible and Incompatible Crosses 

To identify genes involved in the pistil barrier function of the *Ga2-S* locus, we conducted transcriptomic profiling of six different silks: B73 (B), selfed B73 (BB), B73 pollinated by 511L (BG); 511L (G), selfed 511L (GG), and 511L pollinated by B73 (GB). After removing adaptor sequences and filtering low-quality and ambiguous reads, all data were summarized in Table S1. The Pearson *R*^2^ values were between 0.85 and 1.00 for the fragments per kilobase of transcript per million fragments mapped (FPKM) values from the replicates of the six different silks (Figure S1), indicating a significant correlation between the biological replicates. The total average number of clean reads from the B73-based silks were 30,497,892 from B, 31,920,376 from BB, and 30,669,706 from BG, among which 94.47%, 92.05%, and 94.53%, respectively, were readily mapped to the B73 genome, and 85.95%, 84.53%, and 88.43% of which were determined to be unigenes using the B73 *RefGen_v3* [54]. From the 511L-derived silks, the total average numbers of clean reads were 31,936,897 from G, 31,246,441 from GG, and 27,568,918 from GB, among which 77.44%, 76.94%, and 77.78%, respectively, were mapped to the B73 genome, and 69.99%, 70.20%, and 69.84% were determined to be unigenes. Thus, the total number of mapped reads and uniquely-mapped reads were lower from the G, GG, and GB silks than from the B, BB, and BG silks (Table 1). This discrepancy is most likely due to the genomic differences between B73 and 511L. *Ga2-S* inbred line 511L was obtained from MaizeGDB, and a previous report showed that this line probably originated from a line close to Mexican teosinte [6], while comparative analyses between Mo17, B73, and teosinte revealed a high level of diversity [55]. Thus, it is reasonable to speculate that some of the genes in the 511L genome not found in the B73 reference genome might be due to the absence of these genes in B73 genome, or may not have been mapped by our sequencing. Moreover, the pollinated and unpollinated silks of B73 and 511L were grouped together, respectively (Figure S2), suggesting that these two lines are distinct at the transcriptional level. 

**Figure 3:**
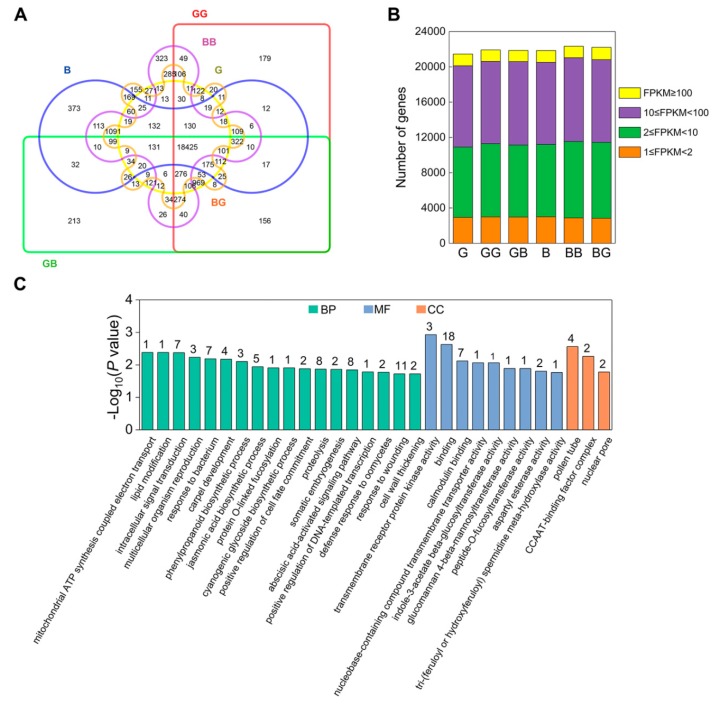
The global patterns of gene expression in all samples. (**A**) Venn diagram of global expression patterns in six silk tissues. (**B**) Distribution of transcript expression levels in four stages (FPKM ≥ 1). (**C**) Overrepresentation of functional categories in GB-enriched genes. The numbers of enriched genes in each gene ontology (GO) term are shown. BP, biological process; MF, molecular function; CC, cellular component.

To identify genes involved in the pistil barrier function of the *Ga2-S* locus, clean reads were normalized using Cufflinks, and the fragments per kilobase of transcript per million fragments mapped (FPKM) values were used to calculate the relative gene expression levels (Table S2) [56]. In total, we detected 25,759 genes in at least one of the six silk tissues using an FPKM value ≥1 as a criterion (Figure 3A and Figure S3A). Among the genes detected, 21,847, 22,329, and 22,210 genes were expressed in the silks of B, BB, and BG, respectively (Figure S3B), whereas 21,453, 21,911, and 21,864 genes were expressed in the silks of G, GG, and GB, respectively (Figure S3C). Of these genes, 18,425 (71.5%) were shared in all silks, 20,217 (87%) were present in all G, GG, and GB silks, and 20,439 (86.1%) were found in the transcriptomes of all B, BB, and BG silks. The expressed genes were classified into four categories based on their FPKM values: extremely low expression (FPKM < 2), low expression (2 ≤ FPKM < 10), medium expression (10 ≤ FPKM < 100), and high expression (FPKM ≥ 100) (Figure 3B, Table S3). No significant differences in the number of genes present in each of the above four categories were detected between the silks of B, BB, and BG, nor between the silks of G, GG, and GB.

### 2.3. The Genes Involved in Signal Transduction Are Active in GB

As shown in Figure 3A, a set of 213 unique genes was specifically expressed in GB silk tissues (Table S4). To gain insights into the function of these genes, a gene ontology (GO) term enrichment analysis was carried out, which revealed that genes related to signal transduction and protein binding were enriched in the GB-unique transcripts (Figure 3C). For instance, two of these GB-specific genes encoded leucine-rich repeat receptor-like protein kinases (LRR-RLKs), Pep receptor-like kinase PEPR1 (GRMZM2G428554), and EFR (GRMZM2G463574), which were annotated with the GO terms “transmembrane receptor protein kinase activity” and “intracellular signal transduction”, respectively, suggesting their possible involvement in gametophyte recognition. In addition, genes annotated as containing a ring finger domain, lesion simulation disease resistance LSD1 (GRMZM2G060057), RING-H2 finger ubiquitin-ligase ATL57 (GRMZM2G469371), and a NB-ARC domain (RPM1 disease resistance genes: GRMZM2G302279, GRMZM2G333659) were also enriched in the GB transcripts. These signal transduction genes may therefore participate in pollen tube recognition and rejection during crossing in the 511L silks.

### 2.4. Analysis of DEGs Induced by Pollination

**Figure 4:**
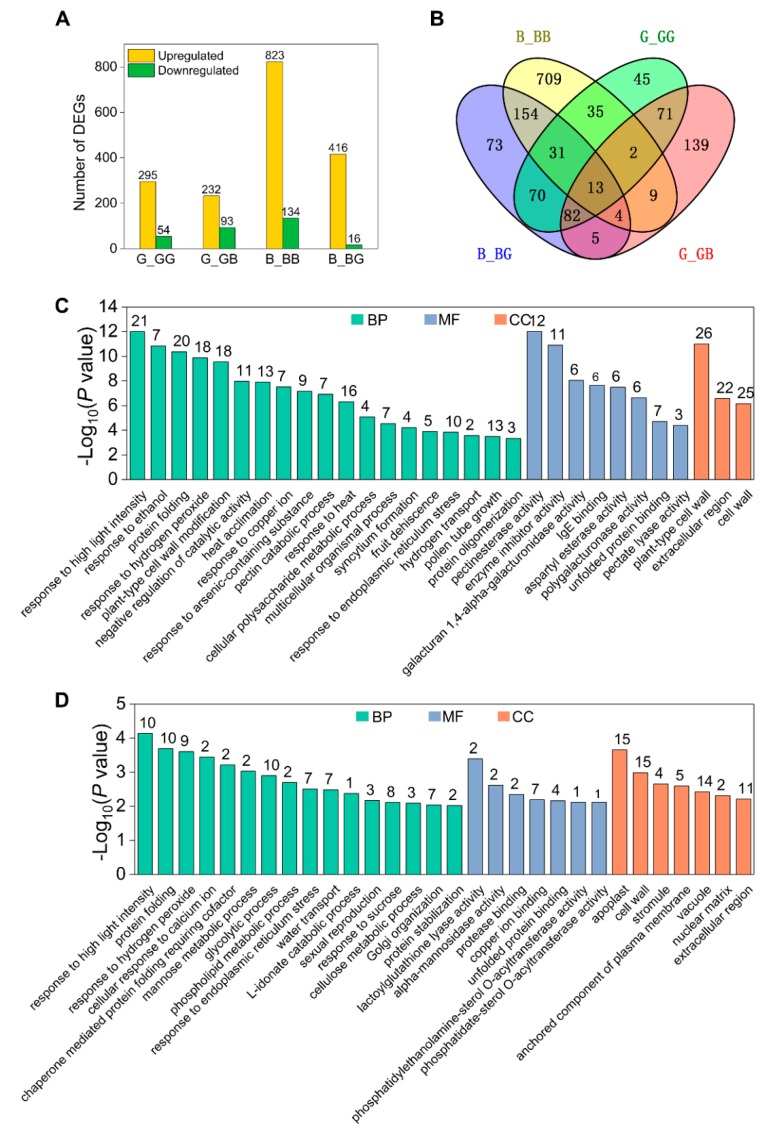
The statistics of differentially expressed genes (DEGs) among different crosses. Expression levels with greater than a two-fold difference between 511L and B73 under different pollinations are shown. (**A**) The number of up- and downregulated genes in four comparisons. (**B**) The overlap of DEGs following the selfing and outcrossing of the two inbred lines. (**C**) GO enrichment of the common response genes and (**D**) specific genes expressed during outcrossing in 511L silks. The numbers of genes in each enriched GO term are shown.

The incompatible pollen tube growth defects in 511L silks initially occur at 2 hap (Figure 2), suggesting that the function of the *Ga2-S* locus may be activated by pollination. To test this, we first identified genes with expression changes induced by selfing and crossing. DEGs induced by pollination were identified in the *Ga2-S* haplotype using a pairwise comparison, using the criteria of a false discovery rate (FDR) ≤ 0.01 and a fold change (FC) ≥ 2 based on their FPKM value. In total, 2063 DEGs were induced by selfing and outcrossing in 511L and B73 in four comparisons: G_GG, G_GB, B_BB, and B_BG, as illustrated in Figure 4A (Table S5). In the B73 silks, the expression levels of 957 and 432 genes were changed upon selfing and outcrossing, respectively. In the 511L silks, fewer DEGs were found to be affected by selfing (349) and outcrossing (325), in comparison with the B73 transcriptome (Figure 4A). Among the four comparisons, only 13 common DEGs were detected, highlighting the prominent differences between B73 and 511L (Figure 4B). Thirty-five common DEGs were detected in B73 and 511L after selfing, among which 34 were collectively upregulated, whereas one was downregulated in G_GG but upregulated in B_BB (Table S6). In the 511L silks, 168 common genes were shared between G_GG and G_GB, which represented the pollination-responsive genes in this cross-incompatibility line. Of these 168 DEGs, 124 were collectively upregulated and 44 were downregulated in 511L plants following selfing and crossing, respectively (Figure 4B, Table S7). Moreover, 139 DEGs were identified during the incompatible crossing of 511L in G_GB, including 92 upregulated and 47 downregulated genes, which were considered to be potentially important for cross-incompatibility (Figure 4B, Table S8).

### 2.5. Cell Wall Metabolism Genes Are Upregulated by Pollination in 511L Silks

As shown in Figure 4C, the GO enrichment analysis revealed 168 genes that were differently expressed in 511L silks following selfing or crossing, which included a set of genes enriched in the molecular function and cellular component categories, with functions such as pectinesterase activity and enzyme inhibitor activity. Remarkably, a set of enzymes was annotated repeatedly with all GO terms related to plant-type cell wall modification and cell wall metabolism, including pectinesterase, enzyme inhibitor, galacturan 1,4-alpha-galacturonidase, polygalacturonase, and pectate lyase. Among these DEGs, eight genes encoded a “pectate lyase superfamily protein”, five genes are annotated as “expansin”, three genes are annotated as “leucine-rich repeat extensin-like protein 1”, and 11 were pectinesterase or pectin methylesterase inhibitors, most of which were upregulated in G_GG, G_GB and B_BG, but not in B_BB. Moreover, genes involved in cell wall loosening or organization were also upregulated, including “glycosyl hydrolase family 9”, “glucan endo-1,3-beta-glucosidase”, and “beta-galactosidase 11” (Table S9). These data suggest that cell wall metabolism may play an essential role during 511L pollination. In addition, among the 44 downregulated genes, the 10 most downregulated genes were annotated as members of the “Hsp20/alpha crystallin family”, which are involved in the response to heat, high light intensity, and protein folding, indicating that Hsp20-mediated signaling may contribute to the *Ga2-S* cross-incompatibility process (Figure 4C, Table S9).

### 2.6. Cross-Incompatibility Pollination Involves Diverse Signaling Pathways in GB

A total of 139 unique DEGs were specifically induced in GB, as shown in Figure 4B. Four *Hsp* genes (*Hsp90-1:* GRMZM5G833699 and *Hsp90-2:* GRMZM2G069651, *Hsp70:* GRMZM2G310431 and GRMZM2G024718), involved in the responses to high light intensity, endoplasmic reticulum stress, hydrogen peroxide, and calcium, were specially downregulated in GB (Figure 4D, Table S9). These genes were enriched in several GO categories, including cell wall metabolism, cellulose metabolism, protease binding, glycolytic process, and vacuole function. Three *GDSL-like lipase* genes (GRMZM2G045215, GRMZM2G158205, GRMZM2G052562), involved in the regulation of plant development and morphogenesis, and two *Phospholipase A2* genes, involved in phospholipid metabolic processes, were upregulated in GB (Figure 4D, Table S9). Phosphoinositides have been reported to play an important role in vesicle trafficking and tip growth. Membrane trafficking is one of the most critical cellular processes for homeostasis and orchestrating pollen tube growth [57,58]. Together, these findings suggest that genes associated with different signaling pathways were induced by 511L outcrossing to mediate cross-incompatibility.

### 2.7. Genetic Background-Dependent DEGs 

**Figure 5:**
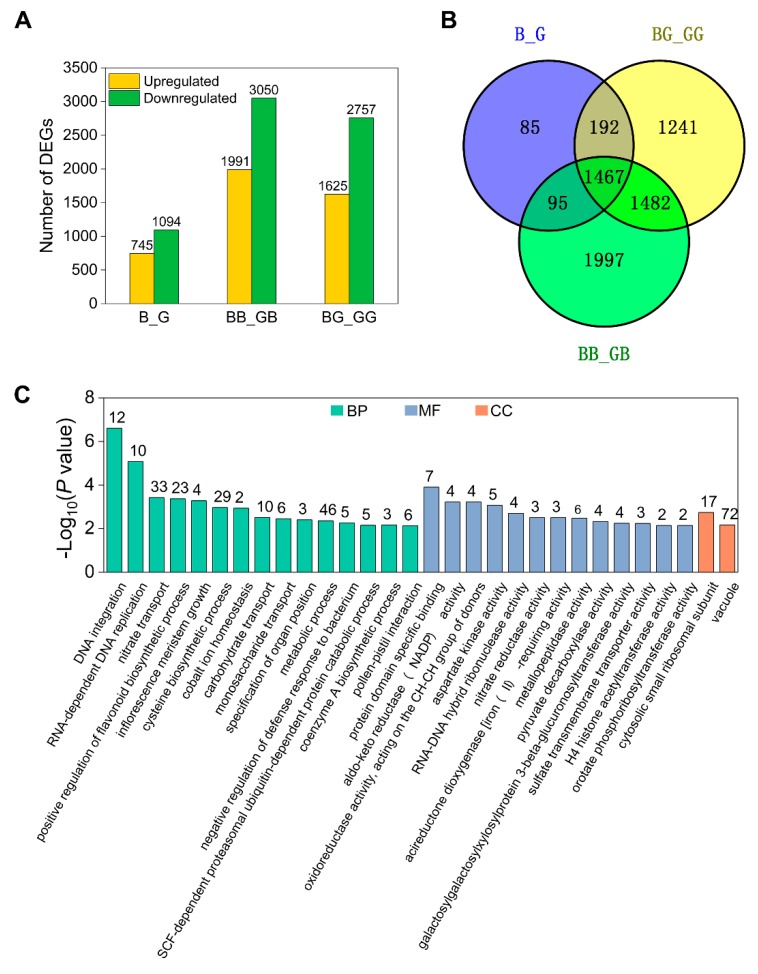
Venn diagram of the DEGs common to 511L and B73. (**A**) Numbers of DEGs in the two genotypes under the same pollination treatment; (**B**) Venn diagram of the DEGs in 511L and B73, before and after pollination with compatible and incompatible pollen; (**C**) GO-term analysis of the 1467 identified DEGs between 511L and B73. The numbers of enriched genes in each GO term are shown.

Given that *Ga2-S* is constitutively expressed in 511L silks, we sought to identify the specific DEGs in different genetic backgrounds under same pollination treatments, including B_G, BG_GG, and BB_GB. Before pollination, 1839 DEGs were identified between the two genotypes (B_G). After pollination, however, we found that 4382 and 5041 genes were responsive to pollination with compatible and incompatible pollen, respectively, in 511L compared with B73 (Figure 5A). DEGs with similar expression patterns were revealed through a hierarchical clustering analysis (Figure S4). For the B_G comparison, only 85 genes were expressed before pollination, and absent after pollination, suggesting that most DEGs might be involved in the pollen–pistil interaction (Figure 5B). A total of 1467 genes were constitutively expressed pre- and post-pollination between the two inbred lines (Table S10), among which 573 genes were upregulated and 894 genes were downregulated in the 511L silks. We focused on the 1467 DEGs detected in the three comparison groups. The common genes were classified into the following GO categories: regulation (RNA-dependent DNA replication and protein domain-specific binding), biological synthesis (positive regulation of flavonoid biosynthetic process, cysteine biosynthetic process, and metabolic process), and nutrition supply (carbohydrate transport and nitrate reductase activity), as shown in Figure 5C. These results suggested that the DEGs in response to pollination were dependent on genetic background.

### 2.8. Functional Network Analysis of Identified DEGs Potentially Involved in UCI

**Figure 6:**
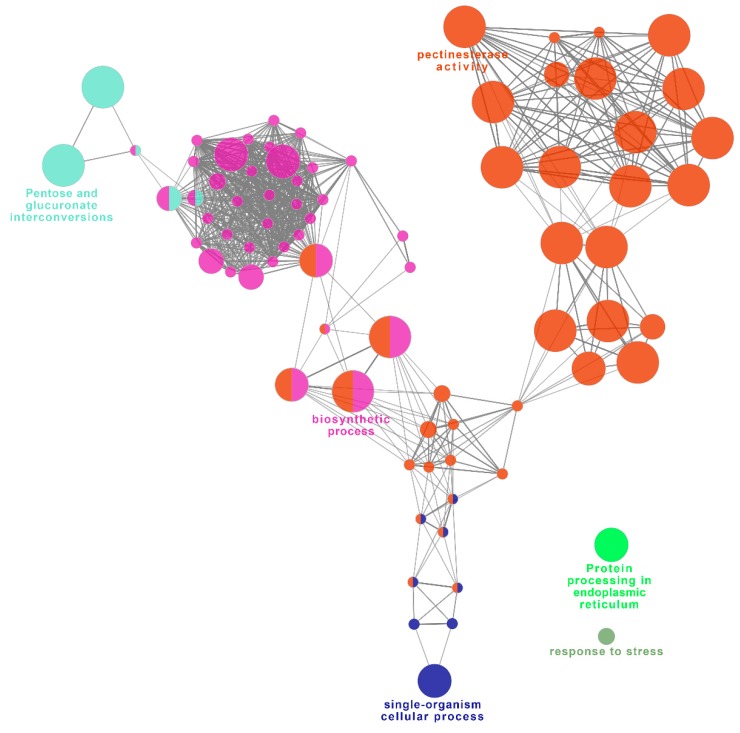
Pathway network analysis of significant gene ontology (GO) terms among candidate genes involved in unilateral cross-incompatibility (UCI). The size of the node circle represents the term enrichment significance (*p* ≤ 0.05). The relationship of grouped network is linked by kappa score level (≥0.3) and the labels of most significant terms are shown. The color of nodes indicates differential function groups.

To further explore the candidate genes involved in UCI, the above-identified genes possibly related to *Ga2-S* to prevent fertilization were further analyzed by constructing a GO/pathway network. Using the ClueGO plugin with a cutoff criteria (kappa score threshold ≥ 0.3) [59], the functional interactions of the representative DEGs were annotated by functional terms and established network interactions. Ninety-nine genes were enriched in several molecular and biological processes, mainly the pectinesterase activity (Figure 6, red nodes), pentose, and glucuronate interconversions (Figure 6, light blue nodes), as well as others in biosynthetic processes (Figure 6, purple nodes), and protein processing in endoplasmic reticulum (Figure 6, green nodes). In particular, genes belonging to carbohydrate metabolism involved in pentose, glucuronate interconversions, and pectinesterase activity were upregulated. Among these genes, pectate lyase superfamily exopolygalacturonases PGA3 (GRMZM2G027782, GRMZM2G160526, GRMZM2G320175, GRMZM2G454608, and GRMZM2G418644) were upregulated in B73 and 511L after pollination (Table S11). The enzymes responsible for the biosynthesis of galactocerebrosides were also enriched, including UDP-glycosyltransferase *88A1* (UGT88A1: GRMZM2G122072 and GRMZM2G417945) and UDP-glycosyltransferase *72B1* (UGT72B1: GRMZM2G162783) (Table S11). These results indicated that sugar metabolism plays an essential role in pollen–pistil nutrition communication. Since growing pollen tubes cannot produce nutrients and normally acquire a ceaseless supply of nutrients from surrounding pistil tissues, sugar metabolism and biosynthetic process in silks are crucial processes for pollen–pistil interaction. Meanwhile, another noteworthy sugar related metabolic pathway was pectin and cellulose metabolism (Figure 6). Several genes involved in pectin modification and cellulose metabolism were upregulated, such as *PME45* (GRMZM2G137676), *PME43* (GRMZM2G128549), *PMEI* (*IQ-DOMAIN 14*: GRMZM2G482245, GRMZM2G167149, GRMZM2G078804, and GRMZM2G462635), and cellulose mannan endo-1,4-beta-mannosidases (MAN7, GRMZM2G055585; MAN2, GRMZM2G140201) (Table S11). The main compositions of plant cell wall are pectin, cellulose, hemicellulose polysaccharides, and some proteins [60,61]. Given that cell wall recombination and modification have been shown to be critical for pollen tube growth and in maintaining tip growth of pollen tubes, our data suggested that those genes are likely associated with the regulation of pollen–pistil interaction in UCI. 

### 2.9. Validation of RNA-Seq Data by qRT-PCR

**Figure 7:**
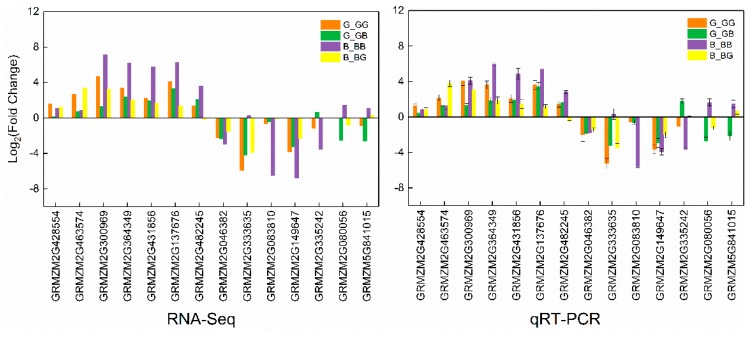
Validation of RNA-Seq data using qRT-PCR. Correlation of expression levels of 12 selected DEGs derived from log_2_ [fold change] in G_GG, G_GB, B_BB, and B_BG pairwise comparisons were determined by linear fitting the RNA-Seq and qRT-PCR data. Error bars represent the SD (*n* = 3).

To validate the expression level of candidate DEGS revealed by RNA-Seq, we selected four cell wall modification genes, three genes encoding LRKs and seven downregulated stress related genes for cross-incompatibility in 511L and B73 reaction. The gene expression values determined using quantitative real-time PCR (qRT-PCR) and the values were transformed into log_2_ [FC] (FC; fold change) and compared with the RNA-Seq data. A relatively higher correlation among the expression levels was obtained by a Pearson correlation coefficient (all at *R*^2^ ≥ 0.7695), as shown in Figure S5. qRT-PCR data displayed the highly similar pattern to that of RNA-Seq (Figure 7), which suggested the reliability of RNA-Seq in identifying the genes regulating UCI in maize.

## 3. Discussion

The entire process of pollination consists of a number of successive steps initiated after pollen deposition on the stigma, including pollen adhesion, hydration, and the germination of the pollen tube. Elongation at the tip is typical for polar pollen tube growth, as pollen tubes first penetrate the intercellular space between the stigma and style, then grow along the nutritious extracellular matrix of transmitting tract tissues towards the ovary. Transmitting tract tissues differ significantly between species, but are thought to consist of specialized cell files associated with the vascular tissue in *Arabidopsis thaliana* [62] and maize [63], while in lily (*Lilium longiflorum* L.), the pollen tubes grow through a hollow pistil [64].

Several previous studies have mapped *Ga1* and *Tcb1*, and reported the allelic functions of these loci present in differently compatible maize lines [8,12,14,15,65], but the mechanisms underlying the crossing barriers involved are still unclear. In this study, we confirmed the cross-incompatibility of 511L, which contains a strong *Ga2-S* allele. An in vivo investigation of the dynamic growth of pollen tubes revealed that their growth in plants expressing the *ga2* allele was blocked at the style elongation stage (Figure 1 and Figure 2), indicating that the cross-incompatibility of *Ga2-S* is caused by defective pollen tube elongation, consistent with the phenotypes observed in the *Ga1-S* and *Tcb1* haplotypes [12,21].

Over the past decade, transcriptional profiling techniques, including microarrays and RNA-Seq, have been widely used for studying male–female interactions during pollination [66,67]; however, the genome-wide transcriptional changes in maize during UCI have not previously been reported. We performed a transcriptomic analysis on maize silk tissues following selfing and outcrossing in two genotypes, 511L (*Ga2-S*) and B73 (*ga2*, wild type). Overall, an average of 93% and 86% reads from the B73 and 511L silk tissues, respectively, were mapped to the B73 reference genome, with this difference highlighting the genetic diversity between the B73 and 511L genetic backgrounds. The total number of transcribed genes we identified, 25,759, is similar to that of a previous study in Zheng58 silks, which identified approximately 23,000 genes expressed in different silks after pollination [68]. 

### 3.1. Genes Involved in Signal Transduction and Defense Are Expressed in Ga2-S Silks

In flowering plants, RLKs are essential for pollen tube guidance, reception, and the rupture of the pollen tube in the ovule [69,70,71]; for example, CrRLK1L members are responsible for pollen tube integrity, and interact with RALF4/19 to prevent the premature rupture of the pollen tube prior to its arrival at the ovule [46,47]. In this work, we identified two LRR-RLKs, PEPR1 (GRMZM2G428554) and EFR (GRMZM2G463574), in the uniquely transcribed genes in the GB silks (Table S9), highlighting the importance of RLKs in *ga2* pollen genotype recognition and rejection. Signal transduction mediated by receptor kinases occurs in a variety of biological processes, such as cell growth, development, and differentiation [72]. In *Brassica rapa*, the papillary cells expressed the *S* receptor kinase gene (SRK) together with the *S* locus glycoprotein gene (SLG) to control SI [42,73]. In the present study, the genes encoding two serine/threonine-protein kinases, HT1 (GRMZM2G028604) and GRMZM2G406601, were found to be specifically expressed in GB, indicating that protein kinase-mediated signal transduction was active during UCI. Previous studies have shown that R genes containing an NB-ARC domain play vital roles in regulating plant resistance, immune responses, and programmed cell death [74]. In this study, genes encoding proteins containing NB-ARC domains, as well as WRKY transcription factors (proteins with WRKY domain, which is normally a 60 amino acid region with conserved WRKYGQK at its N-terminal end), were also found to be induced in GB. Further study is needed to clarify whether these immune response-related genes are indeed involved in the pollen–pistil interaction. 

### 3.2. Cell Wall Metabolism Plays a Crucial Role in UCI

Plant cell walls provide an essential protective barrier, functioning in development and defense. These complex extracellular structures are mainly composed of cellulose, hemicellulose, and pectins, as well as structural proteins, which are typically assembled into a rigid but flexible and dynamically organized network [60,61]. Pectin comprises a highly heterogeneous group of polymers, including the homogalacturonans and rhamnogalacturonans I and II [75]. Previous studies have shown that pectin is biosynthesized in a highly methylesterified form in the Golgi, then secreted into the cell wall [76,77]. A large family of wall-associated PMEs demethylesterifies the pectins in the cell wall by catalyzing the methoxyl groups on the polygalacturonic acid chain, releasing methanol and protons [78]. The exposed free carboxyl groups are capable of crosslinking Ca^2+^ to strengthen the cell wall. At the same time, the pH of the region surrounding the pectin decreases, which could enhance the enzymatic activity of the polygalacturonases and pectate lyases. These enzymes likely contribute to cell wall loosening and facilitate its extension during pollen tube growth [61,79]. PMEI normally suppresses the function of PMEs, thus balancing the degree of pectin methylesterification and controlling cell wall elasticity [36,80]. VANGUARD1 (VGD1) was the first pollen grain- and tube-specific PME to be identified in *Arabidopsis thaliana*, and the altered expression of *VGD1* led to abnormal pollen tube growth [81]. Similarly, altering the expression of two other pollen-specific PME genes, *PME48* and *QRT1*, affected pollen germination and development [82,83]. In this study, we found that pollination altered the expression of different genes in 511L plants during selfing and outcrossing (Figure 4B, Table S5). Among these, a set of genes associated with cell wall pectin metabolism, including Pectinesterase 45 (GRMZM2G137676) and IQ-DOMAIN 14 (GRMZM2G482245), were upregulated in 511L but not in the B73 silks, in addition to many cell wall modification-related genes, such as *PME43* (GRMZM2G128549), *PMEI* (GRMZM2G167149, GRMZM2G078804, and GRMZM2G462635), and cellulose mannan endo-1,4-beta-mannosidase 1 (MAN7, GRMZM2G055585; MAN2, GRMZM2G140201) were particularly enriched by ClueGO analysis (Figure 6, Table S11). β-Mannanase, an extracellular enzyme, has hemicellulase activity or the activities of both hemicellulase and cellulose [84], which belong to the slide wall of pollen tube. These results indicate a potential role for these genes in cross-incompatibility in *Ga2-S* silks. Furthermore, during tip-growth of pollen tube in pistil, the pollen tube elongation depended on the surrounding nutrients supply. Pentose and glucuronate interconversions and biosynthetic process were dramatically enriched as revealed by ClueGO functional network analysis. UDP-glycosyltransferase 88A1 (UGT88A1: GRMZM2G122072 and GRMZM2G417945) and UDP-glycosyltransferase 72B1 (UGT72B1: GRMZM2G162783) were upregulated after pollination in both B73 and 511L (Figure 6, Table S11), which could mediate the transfer of glycosyl residues from activated nucleotide sugars to acceptor molecules (aglycones), thereby regulating bioactivity, solubility, and transport acceptors within the cell and throughout the organism [85]. Thus, this might impact sugar metabolism and indirectly cause the blocked pollen tube growth. Additionally, the expansins, a large family of cell wall-loosening proteins, are involved in cell enlargement and other developmental processes, such as cell wall modification [86]. In most cases, the overexpression of *expansin* genes can stimulate plant cell growth [87], and downregulation of the expression of *expansins* suppresses growth and development [88]. In our study, the expression levels of five *expansin* genes changed after pollination in 511L; for example, *expansin-B10* (EXPB10, GRMZM2G127106) was upregulated in 511L, but not in the B73 silks, suggesting a role for this gene in silk/pollen tube cell wall extension. Moreover, our results indicate that the regulation of pollen tube apical growth during pollen tube elongation was probably controlled by cell wall-related factors derived from the silks. The differences in the expression of genes involved in cell wall modification and remodeling between the *ga2* and *Ga2-S* silks may contribute to the prevention of pollen tube growth during UCI in *Ga2-S* silks.

### 3.3. The Utilization of Ga2-S in Maize Breeding

Hybrid maize lines are popular for their increased yields. Controlling the pollination of the parental inbred lines is a key to maximizing the genetic purity of the hybrid seeds, particularly for sweet, waxy, and genetically modified maize. Commercial seed production plots are normally geographically isolated from each other by at least 500 m, and temporally isolated by about 10 days; nonetheless, pollen contamination and outcrossing are still largely unavoidable [89,90]. The *Ga*-mediated cross-sterile system provides the potential of avoiding adventitious pollen. Previously, *Ga1-S*, *Tcb1-S*, and *Ga2-S* were reported to facilitate cross-incompatibility in conventional dent and flint maize [6,8,18,91,92]. More recently, the JKN2000 (*ga1 ga1*) parental inbred lines were crossed with SDGa25 (*Ga1-S*/*Ga1-S*, as pollen donor), and the F1 plants were subsequently backcrossed for multiple generations and self-crossed to establish a homozygous JKN2000 (*Ga1-S*/*Ga1-S*) hybrid, which displayed a significantly reduced risk of cross-fertilization [14]. Despite the successful utilization of *Ga1* in the cross-incompatibility system, it still lacks sufficient genetic resources to prevent outcrossing; therefore, the incorporation of *Ga2* will provide an additional strategy to control cross-fertilization.

In conclusion, we demonstrate that incompatible pollen grains can undergo the early processes of adhesion, hydration, germination, and penetration, however, pollen tube elongation is blocked in the *Ga2-S* silk barrier. Through a comprehensive transcriptome analysis, large numbers of genotype-dependent DEGs were found to be induced by pollination, revealing a complex regulatory network in pollen–pistil communication. Specifically, signal transduction and cell wall metabolism related processes, such as pectin modification and sugar metabolism were most significant responses upon pollination in UCI, including LRR-RLKs, PME/PMEI, polygalacturonases and pectate lyases, UDP-glycosyltransferases, and expansins. Pectin, a major component of the cell wall, likely plays a vital role in the extension of the pollen tube tip. These findings will not only help us to understand the molecular mechanisms underlying *Ga2*-mediated UCI, but also provide potential genetic resources for controlling pollination during the production of hybrid seeds.

## 4. Materials and Methods

### 4.1. Growth Conditions and Evaluation of Cross-Incompatibility in Maize

The maize (*Zea mays* L.) inbred line 511L, containing the *Ga2-S* locus, was obtained from the MaizeGDB stock center [93]. The other maize inbred lines used in this study were provided by IGDB (Institute of Genetics and Developmental Biology, Chinese Academy of Sciences). The cross-compatibility of 511L with SDG25 (*Ga1-S*), as well as with the Chinese elite inbred lines Zheng 58, Chang 7-2, L75, Z1, JKN2000F, JKN2000M, Huangzao4, L26, B8-2, Qi319, and B73, was examined. Seed set was also determined by pollinating 30 de-tasseled 511L plants with B73 pollen during the flowering peak (three biological repeats). Seed setting was evaluated post-harvest. All pollination tests were conducted in the Experimental Station in Beijing, China (39°9′ N, 11°3′ E). 

### 4.2. In Vivo Pollen Tube Staining and Growth Analysis

In vivo pollen tube growth was examined using the aniline blue staining method [12,63], with some modifications. At least three individual plants and 30 silks in the crosses 511L × 511L and 511L × B73 were used. Pollinated silks were collected from the entire ear at 0.5, 2, 5, 10, and 20 hap, then soaked overnight in FAA solution (85:10:5 *v*/*v*/*v*, 95% ethanol/formaldehyde/acetic acid) at 4 °C. The fixed silks were rehydrated in 50% and 25% ethanol for 3–5 min, and washed three times with 0.1 M potassium phosphate buffer. Subsequently, the samples were incubated in 8.0 M sodium hydroxide solution for 2 h and washed as above, with subsequent staining in 0.1% (*w*/*v*) hydrosoluble aniline blue (dissolved in 0.1 M potassium phosphate buffer) for 8 h. The pollen tube entry point on the silk surface was regarded as the starting point for growth. Silks with consistent lengths were examined in a relatively dark room and mounted on a slide for microscopic analysis (Olympus BX58, Olympus Corporation, Tokyo, Japan) under UV excitation (wavelengths 340 nm to 488 nm), facilitating the measurement of the pollen tube lengths. 

### 4.3. Tissue Harvest for RNA-Seq Analysis

Silks were collected for RNA-Seq analysis during the flowering peak, from around 9:00 to 12:00. Fresh, mature pollen of 511L and B73 was collected for use either in self-crossing (511L × 511L, GG; B73 × B73, BB) or in hybridization (511L × B73, GB; B73 × 511L, BG). Pollinated silks were collected at 3 hap, when significant differences in pollen tube growth occurred between the compatible and incompatible crosses. Two biological replicates were collected per cross. To ensure the reliability of replicates, only the samples with the Pearson *R*^2^ values higher than 0.85 by QC (Quality Control) were used for subsequent sequencing. To reduce pollen contamination, excess pollen was removed from the silks by washing them three times in distilled water. Non-pollinated silks (511L silks, G; B73 silks, B) were collected as the control group. All tissues were immediately frozen in liquid nitrogen following collection and stored at −80 °C for RNA extraction. 

### 4.4. RNA-Seq Library Construction and Sequencing

Total RNA was isolated using the TRIzol reagent (Thermo Fisher Scientific, Waltham, MA, USA) [94]. To eliminate any residual genomic DNA, a total 5 μg RNA per sample was incubated with DNase I (Roche Life Science, Penzberg, Germany) in an RNase-free solution for 30 min at 37 °C. The mRNA was isolated from the total RNA for fragmentation, cDNA synthesis, and PCR amplification, to generate the cDNA libraries following the Illumina RNA Sequencing Protocol. The quality of RNA extraction was assessed by ND-1000 spectroscopy (Thermo Scientific, Waltham, MA, USA) with concentration greater than 60 ng/μL and OD260/280 between 1.8 and 2.2, and OD260/230 greater than 2.0. The quality of library construction was examined by an Agilent 2100 Bioanalyzer with RNA integrity number (RIN) greater than 8 and 28S/18S value greater than 1. Subsequently, sequencing was performed by the Berry Genomics Company (Beijing, China) on an Illumina HiSeq 2500 V4 platform, following the manufacturer’s recommendations. All 125-bp paired-end reads were collected, and each sample produced no less than 4.0 Gbp of data, with a Q30 quality score >83%.

### 4.5. Sequencing Reads Analysis

The raw data (reads) were filtered using the standard Illumina pipeline, with additional filtering steps to identify the adapters. To obtain the clean data (clean reads) with good quality, the reads containing adapter, poly-N, and low-quality reads were abandoned. All data were collected and aligned to the maize reference genome sequence (B73 *RenfGen_v3*) [95] using TopHat2 criteria (--read-mismatches 2 --read-edit-dist 2 --max-intron-length 5,000,000 --library-type fr-unstranded --GTF genome.gtf --mate-inner-dist 40 --solexa1.3-quals, others with default parameters) following the preprocessing steps [54,96]. The sequence alignments were analyzed using Cufflinks software, assembling into empirical transcripts, and the FPKM was calculated to evaluate the transcript abundances using TopHat and Cufflinks. Gene expression pattern analyses were conducted using the BMK Cloud platform (Biomarker Technology Company, Beijing, China) [97].

### 4.6. DEGs and GO Analysis

Pairwise comparisons were performed on the RNA-Seq data using the DESeq R package (1.10.1), to identify DEGs between the silks pollinated with compatible and incompatible pollen [98]. A two-fold change in gene expression (FPKM) and a FDR <0.01 were regarded as the criteria for differential expression, according to the criteria by others [99,100]. Hierarchical clustering was conducted using Spotfire DecisionSite 8.1 (Spotfire Inc., Palo Alto, CA, USA) [101]. A GO enrichment analysis of the DEGs was conducted using the AgriGO analysis tool [102,103], which was assigned to divide the genes into three categories by their function: molecular function, cellular component, or biological process. A *p*-value < 0.05 was considered to reflect a significantly enriched GO term. 

Using plugin ClueGO of Cytoscape [59], DEGs in the six treatments were functionally annotated using gene ontology (GO) terms for GO/pathway term network analysis and visualized in Cytoscape [104]. Only terms/pathways that were significantly enriched (*p*-value < 0.05, right-sided hypergeometric test with Benjamini–Hochberg correction of false discovery rate) were included in the analysis. The minimum connectivity of the pathway network was created by initially defined groups based on kappa score threshold ≥0.3. The final groups are fixed or randomly colored and overlaid with the network.

### 4.7. qRT-PCR Validation of RNA-Seq Data

Fourteen candidate genes with putative functions were selected and validated by qRT-PCR to confirm the results of RNA-Seq. Total RNA was extracted from tissues of six silks derived from a variety of crosses in 511L and B73 (B, BB, BG, G, GG, GB) by TRIzol reagent (Thermo Fisher Scientific) [94], and the quality control of RNA was conducted as same as in Section 4.4, and treated with DNase I as above. Total RNA (≤1.0 μg) was used as a template to synthesize cDNA, using Thermo Scientific™ Maxima™ H Minus Reverse Transcriptase (RT) kit with, and oligo(dT)18 primer following the manufacturer’s instructions. The synthesized cDNA was diluted 10 times with sterile distilled water before being used for qRT-PCR analysis. Each qRT-PCR reaction consisted of 2 μL template cDNA, 1 μL each of the forward and reverse primers (50 pmol), 10 μL 2× SYBR Green mix (Roche, Penzberg, Germany), and 7 μL RNA-free deionized water. The qRT-PCR amplification was performed on a LightCycler 480 II (Roche Life Science), using the following program: 95 °C for 10 min; followed by 40 cycles of 95 °C for 15 s, 60 °C for 10 s and 72 °C for 10 s; and a final extension step of 72 °C for 10 min. The expression of glyceraldehyde-3-phosphate dehydrogenase (*GAPDH*), used as a normalized internal reference, was confirmed by a semi-quantitative PCR reaction using Taq DNA polymerase (TaKaRa, Tokyo, Japan), and a qRT-PCR with all most equal expression levels in all six samples (Supplemental Figure S5A,B). The relative expression levels were calculated with optimized comparative Ct value as previously described [105]. All genes specific primers used for qRT-PCR are designed by Primer Premier 5 software (PREMIER Biosoft, Indore M.P., India) and listed in Table S12. Each experiment was conducted with three technical replicates using three biological samples.

## Figures and Tables

**Table 1:** Summary of mapping reads based on the B73 *RefGen_V3* genome.

	B	BB	BG	G	GG	GB
**Total reads**	30,497,892	31,920,376	30,669,706	31,936,897	31,246,441	27,568,918
**Total mapped (%)**	28,814,261 94.47%	29,381,957 92.05%	28,992,358 94.53%	24,751,691 77.44%	24,027,406 76.94%	21,441,071 77.78%
**Unique mapped (%)**	26,205,774 85.95%	26,984,829 84.53%	27,122,861 88.43%	22,377,204 69.99%	21,930,700 70.20%	19,252,301 69.84%
**Gene numbers**	21,847	22,329	22,210	21,453	21,911	21,864

B, unpollinated silks of B73; BB, self-crossed silks of B73 at 3 hap; BG, silks of B73 pollinated with 511L pollen at 3 hap; G, unpollinated silks of 511L; GG, self-crossed silks of 511L at 3 hap; GB, silks of 511L pollinated with B73 pollen at 3 hap.

## Supplementary Materials

Supplementary materials can be found at http://www.mdpi.com/1422-0067/19/6/1757/s1.
