# Supplementary material for: Transcriptome Analysis Provides Insight into the Molecular Mechanisms Underlying gametophyte factor 2-Mediated Cross-Incompatibility in Maize
Source: Int J Mol Sci. 2018 Jun 13;19(6):1757. doi: 10.3390/ijms19061757 (PMC6032218; doi:10.3390/ijms19061757)
Supplement: Supplementary file 1 [file ijms-19-01757-s001.zip › ijms-287360-SI proofreading-WM/ijms-287360-supplementary-proofreading.docx]

# Supporting Information legends

**Figure S1.** Pearson correlation of different samples in the RNA-Seq libraries


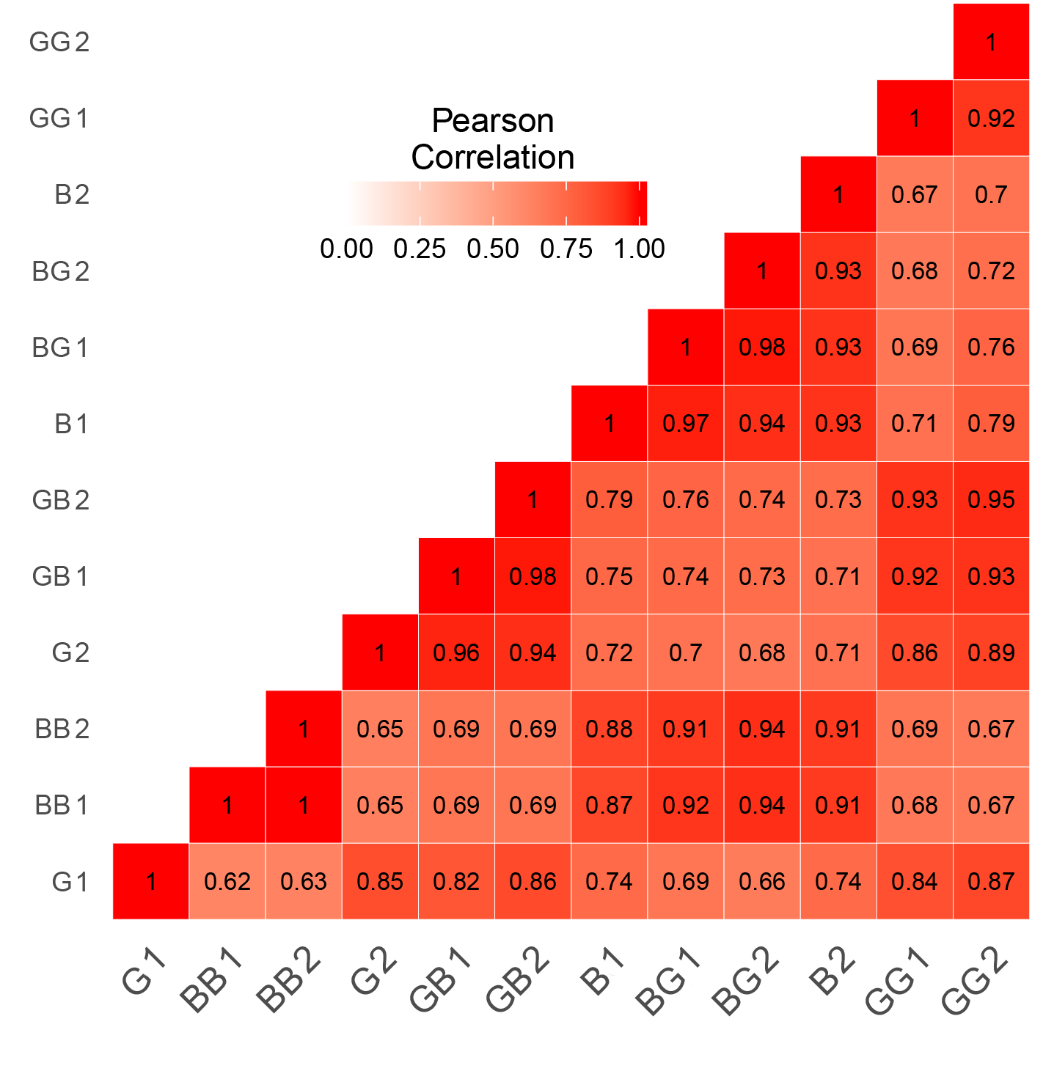


**Figure S2.** Hierarchical clustering of silk transcriptomes


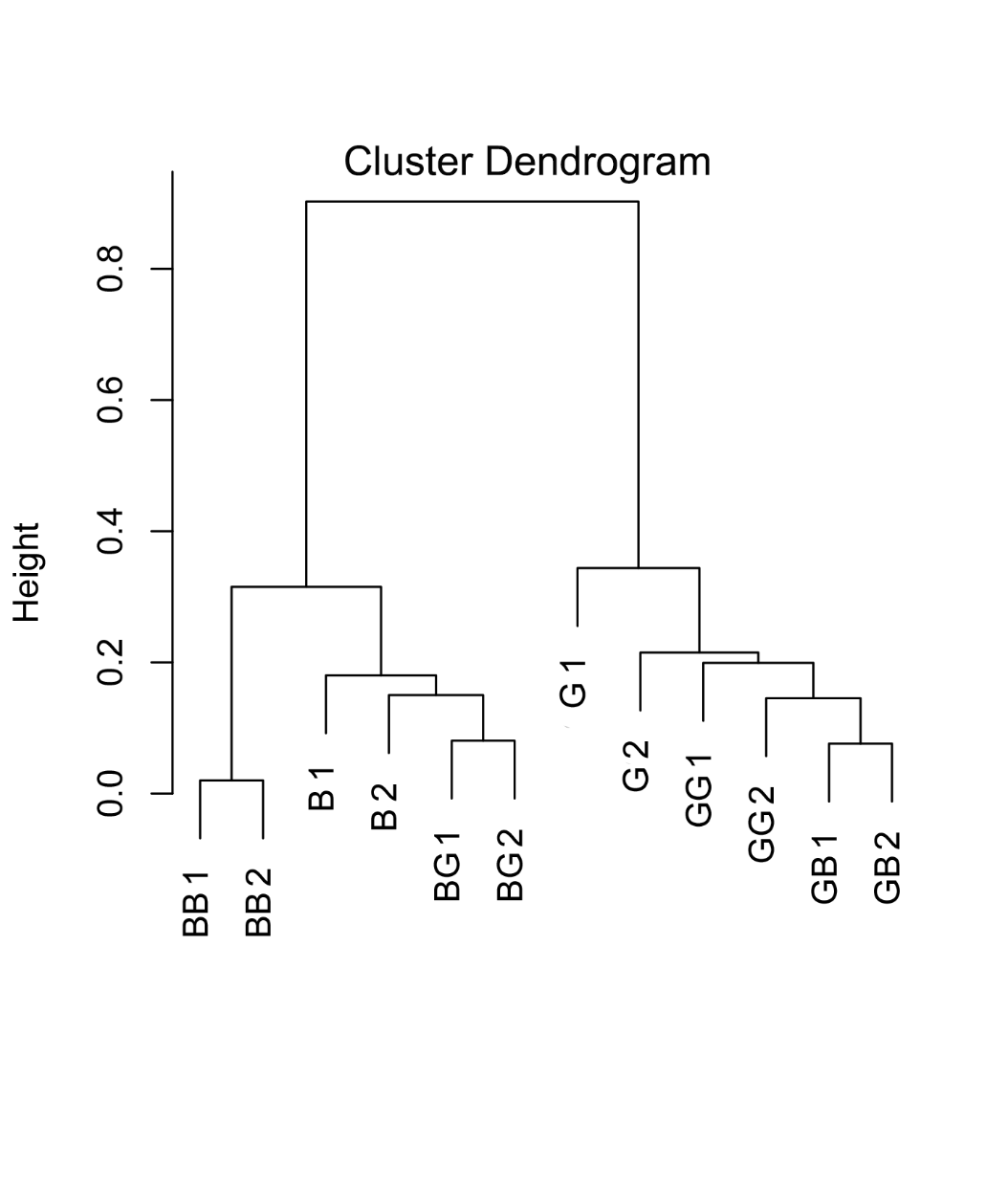


**Figure S3.** Gene expression analysis of silk tissues from compatible and incompatible crosses. **(A)** The number of genes expressed in at least one tissue. In total, 18,425 genes were found in all six silk tissues, 7,334 genes were expressed in more than one tissue, and 1,514 genes were expressed in only one tissue. **(B and C)** The overlapping genes expressed in B73 **(B)** and 511L **(C)** silk tissues after selfing and outcrossing.


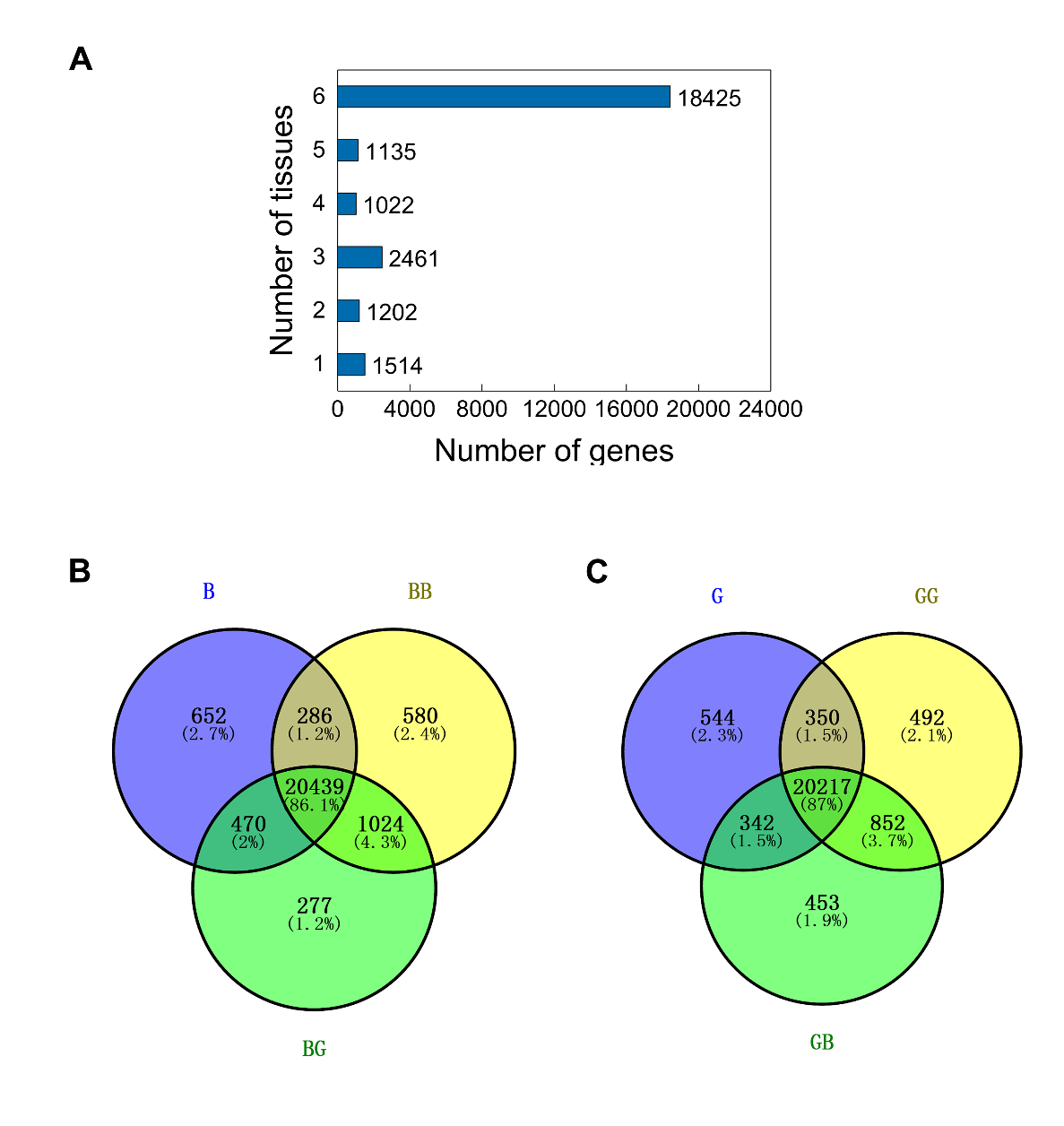


**Figure S4.** Hierarchical cluster analysis of differentially expressed genes (DEGs) for the pairwise comparisons B_G **(A)**, BB_GB **(B)**, and BG_GG **(C)**. Different columns represent different samples, different rows represent different genes, and different colors represent values of (FPKM+1) to indicate different gene expression levels in the samples.


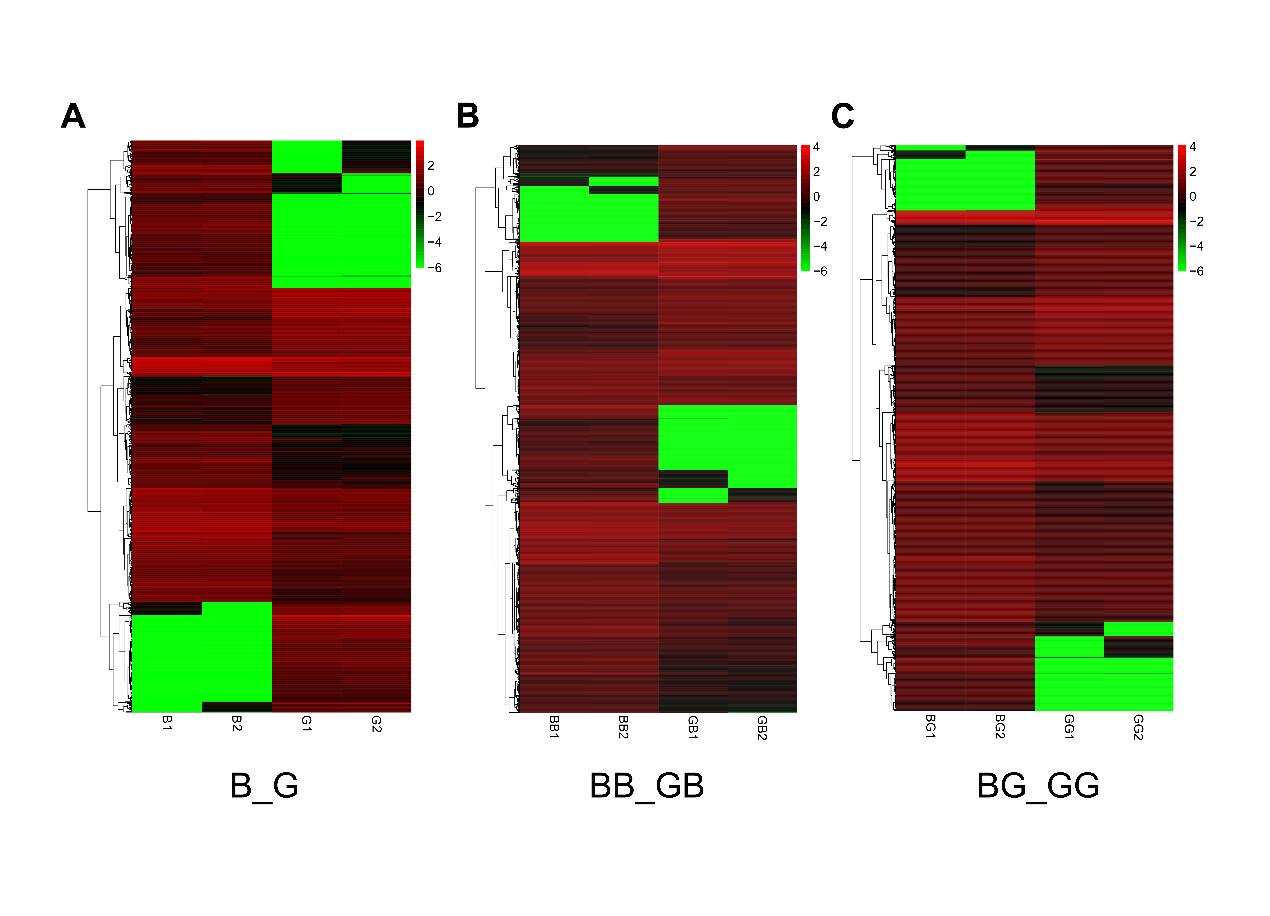


**Figure S5.** Validation of DEGs between 511L and B73 by qRT-PCR under different pollination treatments. **(A)**, expression levels of GAPDH in different tissues by semi-quantitative PCR; **(B)**, fold change of *GAPDH* expression in different tissues by qRT-PCR; **(C-F)**, Correlation of fold change was analyzed between an RNA-Seq (x axis) and qRT-PCR (y axis). Error bars represents the SD (*n*=3).


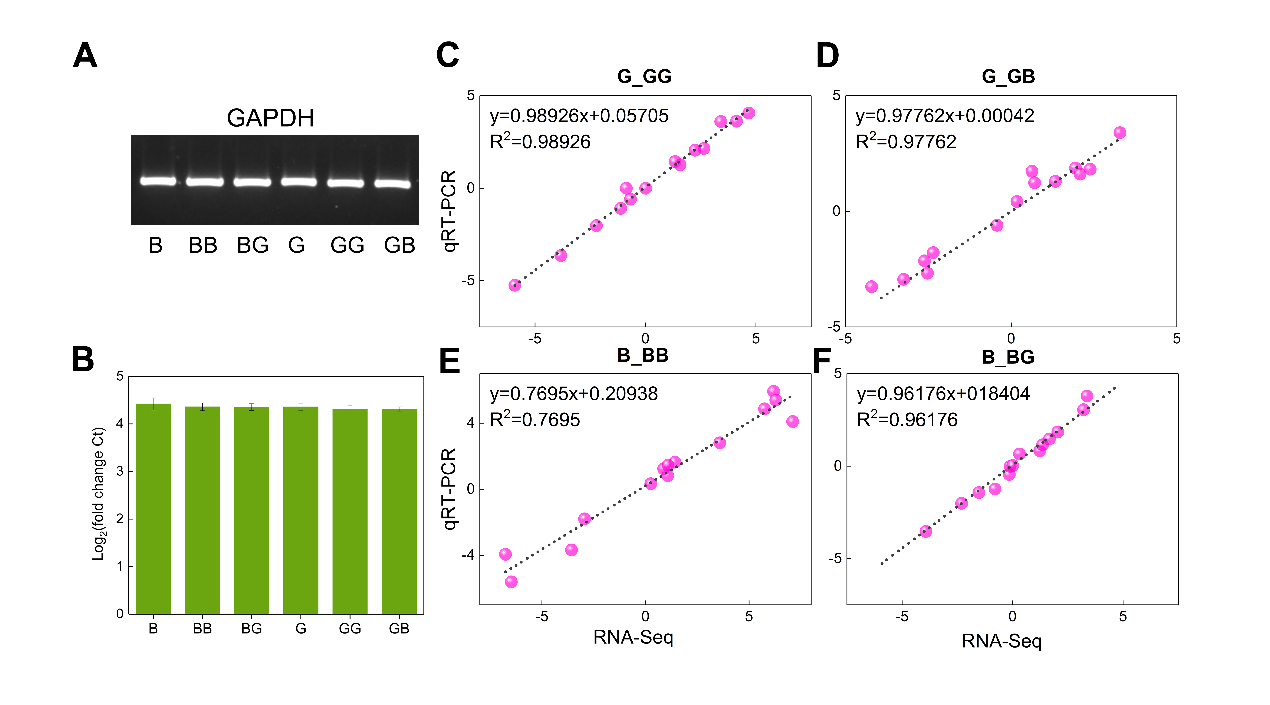


**Table S1.** Distribution of reads sequenced from all tissues in the maize reference genome

**Table S2.** Summary of all gene expression levels based on FPKM in 12 silk tissues

**Table S3.** Statistics of genes expressed at different FPKM levels in six silk tissues

**Table S4.** List of the 213 GB-enriched genes

**Table S5.** Summary of all pairwise comparison DEGs

**Table S6**. List of the shared DEGs between the selfed silks of B73 and 511L

**Table S7.** List of the shared DEGs between the silks of selfed and outcrossed 511L

**Table S8.** List of specific DEGs in response to cross-incompatibility pollination in 511L

**Table S9.** Genes involved in the cross-incompatibility process

**Table S10.** List of shared DEGs in the 511L and B73 lines

**Table S11.** Genes potentially involved in pollen–pistil interaction by ClueGO

**Table S12.** Primers sequences used in this study
